# Supplementary material for: The Prevalence of Depression Symptoms and Their Socioeconomic and Health Predictors in a Local Community with a High Deprivation: A Cross-Sectional Studies
Source: Int J Environ Res Public Health. 2022 Sep 19;19(18):11797. doi: 10.3390/ijerph191811797 (PMC9517619; doi:10.3390/ijerph191811797)
Supplement: Supplementary file 1 [file ijerph-19-11797-s001.zip › ijerph-1817546-supplementary.pdf]

International Journal of
Environmental Research and Public Health

MDPI

Supplementary Materials

The Prevalence of Depression Symptoms and Their Socioeconomic and Health Predic-tors in a Local Community With a High Deprivation Rate in the Example of Residents of Janów Lubelski County in Eastern Poland: A Cross-Sectional Studies

Maciej Polak , Grzegorz Józef Nowicki *, Katarzyna Naylor , Robert Piekarski and Barbara Ślusarska

Table S1. The relationship between the selected socio-economic and health variables and the risk of depression symptoms in the study group when the points were divided into four categories in the PHQ-9 questionnaire.

Variables	Patient Health Questionnaire (PHQ-9)									
	Female (n = 2 201)				p	Male (n = 1 551)				p
	0-4	5-9	10-14	≥ 15		0-4	5-9	10-14	≥ 15	
Age [yes]:	51 ± 8.7	52 ± 8.2	53 ± 7.6	52 ± 7.5	< 0.001	51 ± 8.4	52 ± 7.7	54 ± 7.8	55 ± 8.8	< 0.001
Place of living:										
Rural areas	356 (24.8)	777 (54)	260 (18.1)	45 (3.1)	0.036	380 (35.5)	567 (52.9)	110 (10.3)	14 (1.3)	0.06
Urban areas	218 (28.6)	419 (54.9)	106 (13.9)	20 (2.6)		199 (41.5)	231 (48.1)	40 (8.3)	10 (2.1)	
Marital status:										
Married	506 (26.4)	1 044 (54.5)	311 (16.2)	56 (2.9)	0.35	523 (37.8)	716 (51.8)	128 (9.3)	16 (1.2)	0.002
Single (bachelor/ bachelorette)	37 (29.6)	61 (48.8)	22 (17.6)	5 (4)		50 (34)	71 (48.3)	18 (12.2)	8 (5.4)	
Widow/widower	31 (19.5)	91 (57.2)	33 (20.8)	4 (2.5)		6 (28.6)	11 (52.4	4 (19)	0 (0)	
Education:										
Primary	57 (25.6)	105 (47.1)	50 (22.4)	11 (4.9)	< 0.001	61 (32.1)	94 (49.5)	29 (15.3)	6 (3.2)	0.02
Vocation	170 (25.5)	352 (52.8)	124 (18.6)	21 (3.1)		258 (35.7)	380 (52.6)	76 (10.5)	9 (1.2)	
Secondary	173 (22.3)	453 (58.4)	127 (16.4)	23 (3)		174 (40.7)	220 (51.4)	29 (6.8)	5 (1.2)	

University	174 (32.5)	286 (53.5)	65 (12.1)	10 (1.9)		86 (41)	104 (49.5)	16 (7.6)	4 (1.9)	
Smoking status:										
Yes	68 (27.2)	126 (50.4)	50 (20)	6 (2.4)	0.67	107 (31)	108 (52.2)	47 (13.6)	11 (3.2)	< 0.001
No	506 (25.9)	1 070 (54.8)	316 (16.2)0	59 (3)		472 (39)	618 (51.2)	103 (8.5)	13 (1.1)	
Alcohol consumption:										
No or less than once a month	558 (26.1)	1 160 (54.2)	358 (16.7)	64 (3)		475 (39.4)	600 (49.8)	114 (9.5)	16 (1.3)	
Between once a month and once a week	11 (26.2)	27 (64.3)	4 (9.5)	0 (0)	0.67	61 (31.3)	114 (58.5)	19 (9.7)	1 (0.5)	0.001
More than once a week	5 (26.3)	9 (47.4)	4 (21.1)	1 (5.3)		43 (28.5)	84 (55.6)	17 (11.3)	7 (4.6)	
Lives alone:										
Yes	23 (19.8)	56 (48.3)	32 (27.6)	5 (4.3)	0.007	14 (24.1)	769 (51.5)	141 (9.4)	18 (1.2)	< 0.001
No	551 (16.4)	1 140 (54.7)	334 (16)	60 (2.9)		565 (37.8)	769 (51.5)	141 (9.4)	18 (1.2)	
BMI [kg/m²]:										
Norm [18.5 – 24.99 kg/m²]	202 (31.9)	334 (52.8)	78 (12.3)	19 (3)		112 (41.2)	128 (47.1)	30 (11)	2 (0.7)	
Overweight [25 – 29.99 kg/m²]	203 (25.6)	438 (55.2)	141 (17.8)	12 (1.5)	< 0.001	265 (37)	374 (52.2)	65 (9.1)	12 (1.7)	0.56
Obese [≥ 30 kg/m²]	167 (21.9)	417 (54.7)	144 (18.9)	34 (4.5)		200 (35.7)	295 (52.7)	55 (9.8)	10 (1.8)	
Co-morbidities:[#]										
Yes	142 (21.5)	258 (54.2)	134 (20.3)	27 (4.1)	< 0.001	139 (32.6)	216 (50.6)	60 (14.1)	12 (2.8)	< 0.001
No	432 (28.1)	838 (54.4)	232 (15.1)	38 (2.5)		440 (39.1)	582 (51.8)	90 (8)	12 (1.1)	

[#] Comorbidities: hypertension and/or diabetes and/or hypercholesterolemia.
